# A predominant involvement of the triple seropositive patients and others with rheumatoid factor in the association of smoking with rheumatoid arthritis

**DOI:** 10.1038/s41598-020-60305-x

**Published:** 2020-02-25

**Authors:** Cristina Regueiro, Luis Rodriguez-Rodriguez, Raquel Lopez-Mejias, Laura Nuño, Ana Triguero-Martinez, Eva Perez-Pampin, Alfonso Corrales, Alejandro Villalba, Yolanda Lopez-Golan, Lydia Abasolo, Sara Remuzgo-Martínez, Ana M. Ortiz, Eva Herranz, Ana Martínez-Feito, Carmen Conde, Antonio Mera-Varela, Alejandro Balsa, Isidoro Gonzalez-Alvaro, Miguel Ángel González-Gay, Benjamín Fernandez-Gutierrez, Antonio Gonzalez

**Affiliations:** 1 Experimental and Observational Rheumatology and Rheumatology Unit, Instituto de Investigacion Sanitaria, Hospital Clínico Universitario de Santiago (IDIS), Santiago de Compostela, Spain; 2 Rheumatology Department, Hospital Clínico San Carlos, Instituto Investigación Sanitaria San Carlos (IdISSC), Madrid, Spain; 3 Valdecilla Biomedical Research Institute, Hospital Universitario Marqués de Valdecilla (IDIVAL), Santander, Spain; 4 Rheumatology Department, Instituto de Investigación Hospital Universitario La Paz (IDIPAZ), Madrid, Spain; 5 Rheumatology Department, Instituto de Investigación Sanitaria la Princesa, Hospital Universitario de la Princesa (IIS-lP), Madrid, Spain; 6 Immuno-Rheumatology Department, Instituto de Investigación Hospital Universitario La Paz (IDIPAZ), Madrid, Spain; 7 Faculty of Medicine and Dentistry, University of Santiago de Compostela, Santiago de Compostela, Spain

**Keywords:** Rheumatoid arthritis, Risk factors

## Abstract

The major environmental risk factor for rheumatoid arthritis (RA) is smoking, which according to a widely accepted model induces protein citrullination in the lungs, triggering the production of anti-citrullinated protein antibodies (ACPA) and RA development. Nevertheless, some research findings do not fit this model. Therefore, we obtained six independent cohorts with 2253 RA patients for a detailed analysis of the association between smoking and RA autoantibodies. Our results showed a predominant association of smoking with the concurrent presence of the three antibodies: rheumatoid factor (RF), ACPA and anti-carbamylated protein antibodies (ACarPA) (3 Ab *vs*. 0 Ab: OR = 1.99, p = 2.5 × 10^–8^). Meta-analysis with previous data (4491 patients) confirmed the predominant association with the concurrent presence of the three antibodies (3 Ab *vs*. 0 Ab: OR = 2.00, p = 4.4 ×10^–16^) and revealed that smoking was exclusively associated with the presence of RF in patients with one or two antibodies (RF^+^_1+2_ *vs*. RF^−^_0+1+2_: OR = 1.32, p = 0.0002). In contrast, no specific association with ACPA or ACarPA was found. Therefore, these results showed the need to understand how smoking favors the concordance of RA specific antibodies and RF triggering, perhaps involving smoking-induced epitope spreading and other hypothesized mechanisms.

## Introduction

Rheumatoid arthritis (RA) is a systemic autoimmune disease that can be divided in two pathogenic subgroups^1,2^. The largest subgroup comprises the patients presenting RA specific autoantibodies. These antibodies include the rheumatoid factor (RF), which is directed against the Fc of the IgG, and antibodies against some post-translational protein modifications. The best characterized are anti-citrullinated protein antibodies (ACPA), which in the clinic are assayed as anti-cyclic citrullinated peptides or anti-CCP, and the anti-carbamylated protein antibodies (ACarPA), which are not yet analyzed beyond research studies. The antibody positive patients are known as seropositive and they represent more than two thirds of the total, while the remaining are the seronegative patients. The seropositive patients have a clear component of genetic susceptibility and a defined disease story and autoimmune pathogenesis, which are less clear in the seronegative patients^1–5^.

Also, smoking, which is the major environmental risk factor for RA, is specific of seropositive RA^6,7^. In the ACPA positive patients, the risk is potentiated by interaction with the HLA-DRB1 shared epitope (SE)^8–10^. This interaction together with protein citrullination in the bronchoalveolar lavage (BAL) cells of smokers has been at the basis of an influential pathogenic model^11^. According to this model, smoking induces protein citrullination in the lung, which predisposes to the production of ACPA and, subsequently, of RA^11^. This model has been supported by other findings, including the demonstration of increased PAD2 expression in bronchoalveolar lavage (BAL) of smokers^12,13^. However, not all the findings have been consistent. In particular, no correlation between smoking and protein citrullination^12,14,15^ or between smoking and PAD2^15^ have been observed in the lung tissue specimens. Moreover, the production of ACPA in the lung and the presence of lung abnormalities in early RA were directly associated with ACPA^+^ RA without detectable association with smoking in the available studies^16–19^. Also, van Wesemael *et al*. reported that smoking was associated with the concurrent presence of RF, ACPA and ACarPA, rather than with ACPA in three patient cohorts^20^. The novelty and repercussion of these latter results ask for validation through independent replication.

Here, we have replicated and extended the findings of van Wesemael *et al*. in an independent set of six patient cohorts (n = 2253). Our results confirmed the predominant association of smoking with the concurrent presence of the three RA autoantibodies. Also, thanks to the large power afforded by the combined analysis (n = 4491), we discovered that smoking was exclusively associated with RF positivity in the patients without three concordant positive autoantibodies (RF^+^_1+2_). In contrast, we did not find significant associations of smoking with the specific presence of ACPA or ACarPA. These results indicate that smoking promotes pathways leading to the concurrent presence of the three RA autoantibodies and, in its defect, to the production of RF.

## Material and Methods

### Patients and samples

Patients from five Spanish (IDIPAZ, PEARL, IDIS, IdISSC, and IDIVAL) and one Italian (Rome) RA cohorts were considered as replication sets (n = 2253 with complete data). The Spanish data were directly available to us, whereas the information corresponding to the Italian patients was extracted from a publication^21^. Two of the replication collections were early arthritis (EA) prospective clinics (IDIPAZ and PEARL), whereas the remaining four replication cohorts (IDIS, IdISSC, IDIVAL and Rome) were from established RA patients. Entry criteria for IDIPAZ^22^ and PEARL^23^ were: 2 or more swollen joints for less than a year and absence of previous treatment with Disease Modifying Anti-Rheumatic Drugs (DMARD). In addition, the patients with RA according to the 1987 ACR criteria^24^ at 2 years of follow-up and with serum samples and smoking information in the baseline visit (IDIPAZ, n = 243; and PEARL, n = 264) were selected. The established Spanish RA patients (according to 1987 ACR criteria, with serum samples and information on smoking) were from IDIS^25^, n = 470, IdISSC^26^, n = 508, and IDIVAL^27^, n = 459. In turn, the Italian patients (n = 309) were classified according to the 2010 ACR criteria^21^. Smoking information was obtained as never smoker, past smoker or current smoker in response to the written questionnaire given to the patients at recruitment, either at the first visit (all patients in IDIPAZ and PEARL) or at any time (in the remaining cohorts). No information on smoking intensity was available from most patients. All the patients included in this study granted their written informed consent. The study was designed and conducted according to the Declaration of Helsinki, the Belmont Report and the Spanish Law 14/2007 of Biomedical Research. This included the approval of the Hospital Clínico San Carlos Ethics Committee, the Cantabria Ethics Committee, the EAC by the La Paz University Hospital Ethics Committee, the Ethics Committee for Clinical Research of Hospital Universitario La Princesa (Ref. PI-518), and the approval of the study by the Autonomous Research Ethics Committee of Galicia (Ref. 2014/387 and 2017/514).

For meta-analysis, data from the three patient collections (NOAR, EAC Leiden and BARFOT) included in the van Wesemael study (n = 2238 with complete information) were retrieved^20^.

### Anti-CarP antibodies and other RA autoantibodies

Anti-CarP IgG antibodies were assessed by ELISA as previously described^25,28^. IgM-RF was determined by rate nephelometry, whereas ACPA were determined by ELISA. The ACPA were tested as anti-CCP2 with the EDIA enzyme-linked immunosorbent assay kit in all the IDIS and IDIVAL patients and with the Immunoscan RA in all the IDIPAZ and IdISSC patients (both assays from Euro Diagnostica, Malmö, Sweden). The patients of PEARL were tested with Immunoscan RA until October 2010. Afterward, they were assayed with the QUANTA Lite CCP3 IgG and IgA assay (Inova Diagnostics, San Diego, CA).

### Statistical analysis

Results from the different patient cohorts, and from previously reported cohorts^20,21^, were combined by meta-analysis with the R package *meta*^29^. Subgroup meta-analysis comparing EAC and prevalent RA cohorts was done with Review Manager Version 5.3^30^. Smoking habit was considered as ever or never smoker status. In most analyses, the RF^−^ACPA^−^ACarPA^−^ triple negative patients (0 Ab) were used as the reference. Alternative meta-analyses compared other patient subgroups, which are indicated in the text with a combination of the antibody abbreviation (RF, ACPA or ACarPA), the plus and minus superscripts for presence/absence, and the number of antibodies considered (from 0 to 3) as subscripts. In this way, the patients bearing only RF are coded as RF^+^_1_, those bearing RF and a second antibody as RF^+^_2_. See Supplementary Table S1 for the remaining codes. Heterogeneity between cohorts was assessed with the inconsistency parameter I^2^. By default, the fixed effects model was applied for meta-analysis, weighting the contribution of each cohort with the inverse variance method. The random effects model according to DerSimonian and Laird was preferred when heterogeneity was notable (I^2^ > 50) and reported as OR_re_ and p_re_. P values lower than 0.05 were considered statistically significant.

Additionally, we used exploratory analysis on the pooled data across the cohorts for interpretation of the findings. It included graphic representation and classification trees. For the former, we employed a double-decker plot^31^. The classification trees, in turn, were done with the General Classification and Regression Trees module of Statistica (v7.0, StatSoft, Tulsa, OK) that produces an exhaustive and recursive search of the best classification. We considered nine dichotomous variables to classify the patients according to smoking. The variables represented the presence or absence of the antibodies, their combinations and number: RF, ACPA, ACarPA, RF&ACPA, RF&ACarPA, ACPA&ACarPA, one antibody, two antibodies, and three antibodies. The priors were considered proportional to the smoking class sizes, the misclassification costs were taken to be equal for every class, the splits were selected based in the Gini index of node impurity, and no stops were imposed. This procedure searches the minimum number of univariate splits to produce the tree with less misclassified patients without being limited by the high dependence between the classification variables.

## Results

### Replication of the association of smoking with concurrent autoantibodies

The six newly obtained cohorts of RA patients included 2253 patients with complete data (Table 1). Two of the cohorts were prospective EAC (n = 507), the remaining included patients with established RA (n = 1746). About half (43.6–55.1%) of the patients were ever smokers, except in one of the cohorts where the frequency of smokers was notably lower (20.4%). This circumstance is characteristic of the population attending the recruiting hospital, as previously noted^32^. The other critical characteristic for this study, the presence of autoantibodies, varied between the cohorts within the commonly observed range: mean percentages of RF^+^ = 61.7%, ACPA^+^ = 57.7%, and ACarPA^+^ = 34.4%. The patients were divided into four subgroups by the number of antibodies they presented. These subgroups were of comparable size except for the patients with one antibody that were the less abundant: 26.6, 18.8, 28.8 and 25.8% for the groups with 0, 1, 2 and 3 antibodies, respectively.

**Table 1:** Main characteristics of the patients with RA.

Cohort	New cohorts						van Wesemael *et al*.		
	IDIPAZ^a^	PEARL^a^	IDIS	IdISSC	IDIVAL	Rome^b^	NOAR^a^	EAC Leiden^a^	BARFOT^a^
Complete data, n	243	264	470	508	459	309	674	769	795
Ever smoker, n (%)	117 (48.1)	115 (43.6)	96 (20.4)	232 (45.7)	253 (55.1)	167 (54)	432 (63.7)	415 (54)	471 (59.2)
RF positive, n (%)	168 (69.1)	175 (66.3)	281 (59.8)	329 (64.8)	235 (51.2)	202 (65.4)	277 (40.9)	442 (57.5)	448 (56.4)
ACPA positive, n (%)	170 (70)	163 (61.7)	300 (63.8)	251 (49.4)	225 (49)	190 (61.5)	247 (36.4)	404 (52.5)	456 (57.4)
ACarPA positive, n (%)	109 (44.9)	100 (37.9)	141 (30)	158 (31.1)	149 (32.5)	117 (37.9)	182 (26.8)	349 (45.4)	279 (35.1)
**Number of autoAb**.									
0	54 (22.2)	59 (22.3)	111 (23.6)	141 (27.8)	173 (37.7)	62 (20.1)	292 (43.1)	242 (31.5)	263 (33.1)
1	21 (8.6)	45 (17)	95 (20.2)	118 (23.2)	78 (17)	67 (21.7)	167 (24.6)	129 (16.8)	110 (13.8)
2	78 (32.1)	87 (33)	165 (35.1)	127 (25)	93 (20.3)	98 (31.7)	108 (15.9)	128 (16.6)	193 (24.3)
3	90 (37)	73 (27.7)	99 (21.1)	122 (24)	115 (25.1)	82 (26.5)	107 (15.8)	270 (35.1)	229 (28.8)

Analysis of the six new cohorts (n = 2253) that were studied here for the first time and of the three cohorts (n = 2238) from a previous study^20^.

^a^Early arthritis patients.

^b^Data extracted from Pecani *et al*.^21^.

We assessed the relationship of smoking with the patients grouped according to the number of antibodies they presented (Table 2). Only the patients positive for the three autoantibodies were significantly associated with smoking (3 Ab *vs*. 0 Ab: OR = 1.99, p = 2.5 ×10^−8^). In stark contrast with this very significant result, the patients with one or two autoantibodies were not significantly different from the patients without antibodies (Table 2). In addition, the association of smoking with the concurrent presence of the three antibodies was significant not only relative to the patients without antibodies (Table 2), but also relative to the patients with one antibody (3 Ab *vs*. 1 Ab:OR = 1.68, 95% CI = 1.32–2.13, p = 2.2 ×10^−5^, I^2^ = 0%) and with two antibodies (3 Ab *vs*. 2 Ab: OR = 1.61, 95% CI = 1.27–2.04, p = 8.1 ×10^–5^, I^2^ = 0%).

**Table 2:** Association of smoking with seropositive patients with different numbers of autoantibodies in the new patient cohorts^a^.

	0	Number of autoantibodies		
		1	2	3
**IDIPAZ**				
Non-smoker, n^b^	40	7	38	41
Smoker, n	14	14	40	49
**PEARL**				
Non-smoker, n	37	28	51	33
Smoker, n	22	17	36	40
**IdISSC**				
Non-smoker, n	90	60	75	51
Smoker, n	51	58	52	71
**IDIS**				
Non-smoker, n	83	81	138	72
Smoker, n	28	14	27	27
**IDIVAL**				
Non-smoker, n	88	39	41	38
Smoker, n	85	39	52	77
**Rome**				
Non-smoker, n	31	34	45	32
Smoker, n	31	33	53	50
**Summary statistics**				
OR	1 (ref.)	1.19	1.19	1.99
95% CI	—	0.91–1.55	0.94–1.52	1.56–2.54
p	—	0.21	0.15	2.48 × 10^−08^
I^2^, %	—	68.9	56.9	25.9
OR_re_	1 (ref.)	1.22	1.22	1.99
95% CI_re_	—	0.74–2.01	0.84–1.77	1.49–2.64
p_re_	—	0.43	0.30	2.47 × 10^−06^

^a^The table presents the number of patients in each category in the upper part and the summary statistics obtained with meta-analysis in the lower part. The triple negative patients were used as reference (ref.) for the patients with 1, 2 or 3 antibodies.

^b^n = number of subjects; OR = odds ratio; CI = confidence interval; I^2^ = inconsistence; the re subscript indicates the random effects model.

### Combined meta-analysis of the available data

**Figure 1:**
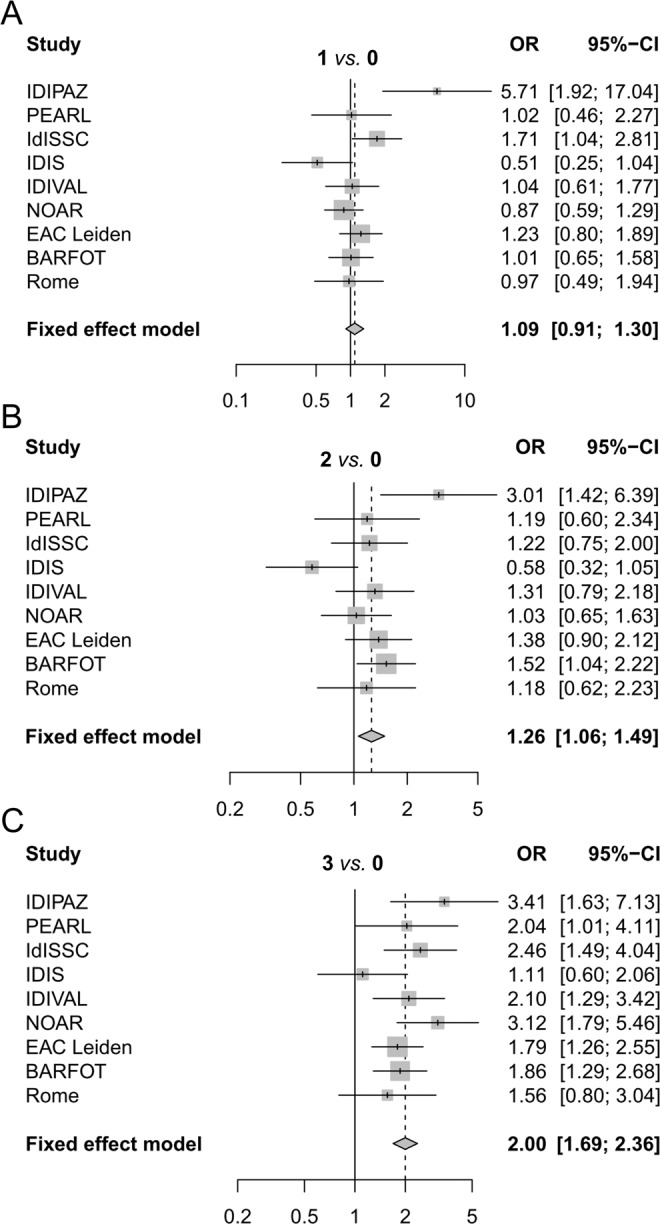
Smoking association with seropositivity for different numbers of autoantibodies in the combined meta-analysis. Forest plots showing the fixed-effect meta-analysis involving the RA patients that were positive for (**A**) one, (**B**) two, and (**C**) three autoantibodies. The patients without antibodies (0 Ab) were taken as reference. The OR corresponding to each cohort (Study) and its 95%-CI are provided and shown by a vertical tick and the length of a horizontal line, respectively. The area of the square around the tick is proportional to the size of the study. Summary OR and 95% CI are given in bold and represented as a vertical dashed line and a diamond, respectively. The OR scale is logarithmic.

**Figure 2:**
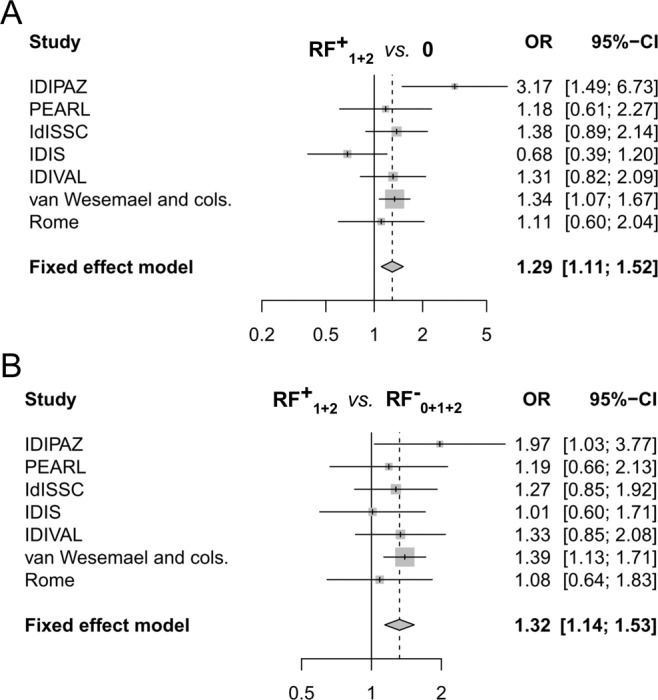
Association of smoking with the presence of RF in the RA patients carrying one or two antibodies. Forest plots showing the comparison of: (**A**) RF^+^ patients that were positive for one or two antibodies (RF^+^_1+2_) with the patients without antibodies (0); and (**B**) the comparison of RF^+^ patients that were positive for one or two antibodies (RF^+^_1+2_) with all the RF^−^ patients (RF^−^_0+1+2_). No cohort-specific information was available for the three cohorts in van Wesemael *et al*. Plots follow Fig. 1 conventions.

We combined the 6 replication cohorts with the 3 cohorts form van Wesemael *et al*.^20^ to a total of 4491 patients with RA. The summary data showed that the association with the concurrent presence of the three antibodies was highly significant (Fig. 1C, 3 Ab *vs*. 0 Ab: OR = 2.00, p = 4.4 ×10^−16^, I^2^ = 17%), and significantly stronger than the observed with the patients carrying two antibodies when directly compared (3 Ab *vs*. 2 Ab: OR = 1.54, 95% CI 1.29–1.84, p = 1.4 ×10^−6^, I^2^ = 12%). Even so, the patients with two concordant positive antibodies were associated with smoking (Fig. 2B, 2 Ab *vs*. 0 Ab: OR = 1.26, p = 0.009, I^2^ = 41%). In contrast, the patients carrying only one antibody were not significantly associated with smoking (Fig. 2A, 1 Ab *vs*. 0 Ab: OR_re_ = 1.12, p_re_ = 0.4, I^2^ = 56%). These associations were not different in the EAC and the prevalent RA cohorts (Supplementary Table S2).

### Association of smoking with the presence of RF in the patients with one or two positive antibodies

The patients carrying RF accounted for the smoking associations in the patients without the concurrent presence of the three antibodies. First, the specific RF association was found in RA patients with only one positive antibody (1 Ab) (Supplementary Table S3). In them, the patients carrying RF (RF^+^_1_) were associated with smoking (RF^+^_1_ vs. 0 Ab: OR = 1.28; 95% CI = 1.03–1.61; p = 0.03), whereas the RA patients carrying other positive antibodies (RF^−^_1_) were indistinguishable from the triple negative patients (RF^−^_1_ vs. 0 Ab: OR_re_ = 1.01; 95% CI = 0.65–1.56; p_re_ = 0.97). In addition, smoking was also associated with the presence of RF in the RA patients carrying two positive antibodies (2 Ab) (Supplementary Table S4). In effect, the patients in whom one of the two positive antibodies was RF (RF^+^_2_) were significantly associated with smoking (RF^+^_2_ *vs*. 0 Ab: OR = 1.30; 95% CI = 1.09–1.55; p = 0.004), whereas the other patients carrying two antibodies (RF^−^_2_) were undiscernible from the triple negative patients (RF^−^_2_ *vs*. 0 Ab: OR = 0.95; 95% CI = 0.64–1.39; p = 0.78). Similar analyses centered on the presence of ACPA or ACarPA did not show any significant association.

A notable finding of the preceding analysis was the very similar association (OR = 1.28 and 1.30) of smoking with patients carrying RF in the patients with only one positive antibody (RF^+^_1_) and with two positive antibodies (RF^+^_2_). This equivalence was confirmed when the associations of smoking with these two subgroups of patients were directly compared (RF^+^_2_ *vs*. RF^+^_1_: p = 0.9). As a consequence, the RF^+^ patients carrying one or two positive antibodies were grouped (RF^+^_1+2_). As expected, smoking was associated with this unique RF^+^ subgroup (Fig. 2A, RF^+^_1+2_ *vs*. 0 Ab: p = 0.001, I^2^ = 45%). An association that was slightly reinforced when all the RF^−^ patients were used as reference (Fig. 2B, RF^+^_1+2_ *vs*. RF^−^_0+1+2_: p = 0.0002, I^2^ = 0%). It should be remarked that the patients without RF in this latter analysis (RF^−^_0+1+2_) included patients positive for ACPA or ACarPA or both these antibodies. The previous associations were not significantly different in the EAC and the cohorts including prevalent RA patients (Supplementary Table S5).

### Global exploratory analysis

We used two exploratory techniques to check if a global analysis of all patient subgroups together was consistent with the sequential analyses in the preceding paragraphs without generating redundant statistical tests.

**Figure 3:**
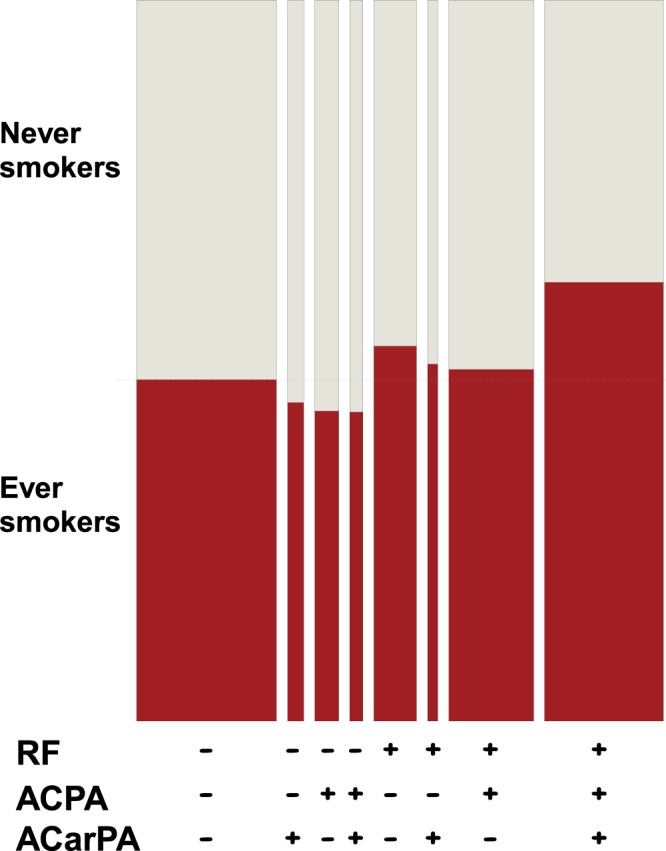
Smoking frequency in the patients stratified by the presence of the three autoantibodies. The double-decker plot divides the patients in rectangles proportional to the frequency in each subgroup. The width is proportional to the size of the antibody-defined patient subgroups, whereas the height of the red rectangles is proportional to the ever smokers within the antibody subgroups.

First, the relative frequencies of ever smokers and never smokers in each of the autoantibody-defined strata were displayed with a double-decker plot. Consistently with the results obtained in the sequential analyses, the smokers were enriched in the RF^+^ patients relative to the corresponding RF^−^ patients in all the strata (Fig. 3).

**Figure 4:**
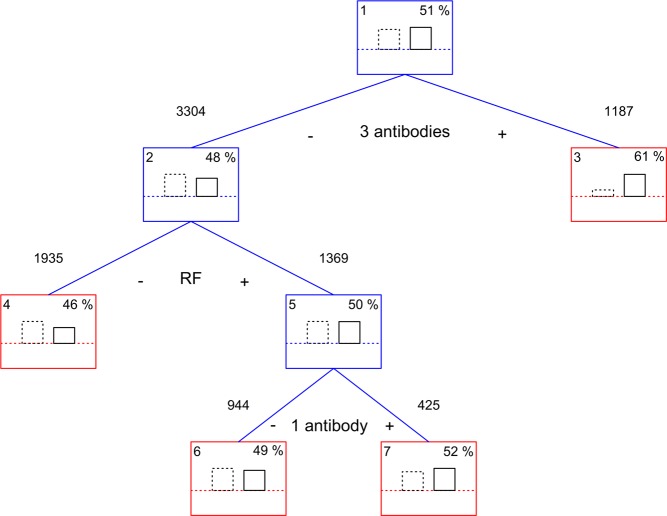
Classification tree of the patients by the presence of antibodies according to smoking. Each node shows its number (upper left corner), the percentage of ever smokers (upper right corner), and an histogram representing the frequency of ever smokers (continuous line) and never smokers (dotted line). Decision nodes are in blue whereas terminal nodes are in red. The splitting conditions are displayed below the node with indication of the value determining each side of the split and the number of subjects sent to the child node.

The second exploratory analysis consisted of an exhaustive search of the best classification tree discriminating ever smokers from never smokers based on the antibodies and their combinations (Fig. 4). The first split of the tree was according to the concurrent presence of the 3 antibodies. The 1187 patients with concordant triple seropositivity (3 Ab) contained 61% of smokers, whereas the remaining patients only 48% of smokers. No other antibody or combination contributed to the classification of the concordant patients. In contrast, two more divisions were observed in the non-concordant patients. The second split was on the presence of RF. The RF^+^ group (RF^+^_1+2_) contained more smokers than the RF^−^ stratum (RF^−^_0+1+2_). The third split was unanticipated and complex. It divided the RF^+^ patients into a smoker-enriched subgroup in whom RF was the only present antibody (RF^+^_1_), and a smoker-depleted subgroup in which RF was present concurrently with other antibodies (RF^+^_2_). This latter division reinforced the exclusivity for RF of the smoking association in the patients with one or two antibodies.

## Discussion

Our main findings have been the predominant association of smoking with the concordant presence of the three RA antibodies, and the exclusive association with the presence of RF in the seropositive patients with one or two antibodies (RF^+^_1+2_). In addition, there was no significant association of smoking with the presence of ACPA or ACarPA. These findings have represented a significant advance. In van Wesemael *et al*.^20^, the association of smoking with RA could represent a gradual increase in the strength of association with the number of antibodies. The evidence presented here excludes this putative mechanism by the demonstration of the exclusive association of smoking with RF independently of the presence or absence of other antibodies in the patients carrying one or two antibodies, and by the clear distinction between the patients with three antibodies as a separate classification to other seropositive patients. This fundamental insight determines the nature of the models aiming to explain the effect of smoking on RA susceptibility. In addition, our analysis has made more understandable the relationship between smoking and the antibodies thanks to the exploratory techniques. They also showed that the results of the statistical tests were faithful to the data.

The originality of our findings is reflected in the absence of any other study analyzing the association of smoking with combinations of the three antibodies included here and in van Wesemael *et al*.^20^. Therefore, we considered the studies assessing the combination of RF and ACPA as antecedents. We have found only reports from four large cohorts^20,33–35^, one of them included in van Wesemael *et al*. but different from the RA cohorts included in our meta-analysis. The four cohorts were large, with ≈9500 healthy Japanese subjects^20^, ≈2000 UK RA patients^33^, ≈1500 USA RA patients^34^, and ≈3600 Swedish RA patients^35^. The three first have shown a significant association of smoking only with the concurrent presence of the two antibodies, not with any of them in isolation. The forth by Hedstrom *et al*. showed a stronger association with the concurrent presence of RF and ACPA, followed by RF and less significantly by ACPA (more on the results of this study below). These results and those of another study with two smaller UK RA collections^36^ are fully compatible with our findings. In addition, the bibliographic search brought to our attention another important fact. None of the studies that support the pathogenic model linking smoking with RA through the production of ACPA has accounted for the association of smoking with the concurrent presence of ACPA and RF^10,11,37–39^ until very recently^35^.

We do not know the mechanism behind the predominant association of smoking with the concurrent presence of RA autoantibodies. However, it is well-known that the status of the RA autoantibodies is much more concordant than at random^25,28,40^. This circumstance reveals the existence of pathogenic mechanisms that are shared by the various antibodies. Some of these mechanisms contribute to epitope spreading, which characterizes the progression of T and B cell responses in autoimmune diseases including the preclinical phase of RA^3–5,41,42^. In effect, the earliest seropositive samples from patients that will develop RA years later often recognize a single epitope, whereas samples taken near the clinical onset recognize multiple epitopes^3–5^. Therefore, smoking could promote concordant seropositivity by broadening and accelerating epitope spreading.

The known factors affecting epitope spreading include the availability of epitope-specific lymphocytes, reflecting incompetent tolerance, and favorable T - B cell interactions and antigen presentation^41,42^. The latter interactions could be boosted by bystander activation, tissue damage and inflammation. Therefore, smoking could promote epitope spreading through these multiple mechanisms. This is possible because the triggering of inflammation and tissue damage, the recruiting and activation of neutrophils, monocytes, and macrophages, and abnormalities in NK, dendritic cells, B cells and many subtypes of T lymphocytes are some of the many effects of smoking on the immune responses^43,44^. The overall balance of this range of actions is an increased predisposition to autoimmunity and the production of autoantibodies^43,44^. Specifically, smoking has been associated with the production of anti-dsDNA in SLE^45^, anti-Jo1 in inflammatory myopathies^46^ and of RF and other autoantibodies in smokers without any autoimmune disease^20,47–50^.

The sharing of immunological mechanisms between the autoantibodies is also the most likely explanation for the correlation between their antibody titers, a correlation that we have also observed in our analyses^25,51–53^. These correlations have been observed independently of the disease stage and, most significantly, to be maintained as parallel titer decreases in response to treatment revealing that they respond similarly to the control of inflammation^51–53^. These correlations lack any specific direction and extend to the thresholds for establishing the positive/negative status (Supplementary Table S6). Therefore, they are unlikely to denote any hierarchy of precedence or causality between the antibodies. Just recently, some of these examples of shared and overlapping immunological mechanisms have been characterized as the convergent pathways model of RA pathogenesis^54^.

The association of smoking with RF in the patients with one of two antibodies suggests that RF could precede other autoantibodies in the smokers that will become RA patients. However, we lack support for this interpretation given the cross-sectional nature of our sample collections. In addition, the studies of preclinical RA samples have been discordant in the order of antibody appearance: IgA RF and IgM RF were the first antibodies in a Swedish cohort^55^, whereas ACPA^4^ and ACarPA^56^ preceded IgM RF in a Dutch cohort, and again IgG ACPA preceded RF (no ACarPA analysis included) in American military^57^. Similarly, the presence of RF in smokers without RA that has been known for more than two decades^49,50^, cannot be taken as evidence of RF preceding the other autoantibodies because recent studies have found also an increased presence of ACPA in smokers without RA^20,58^. In consequence, we will need to wait for new studies to solve this question.

Independently of the order of appearance, we need an explanation for the specific association of smoking with RF. A couple of possible mechanisms have already been proposed. One of them was originally developed to explain the production of autoantibodies in smokers without autoimmune diseases^48^. It starts by the induction of heat-shock protein 70 (Hsp70) expression and of antibodies against Hsp70 by smoking. In the next step, the production of RF is triggered by the two signals given by the Hsp70 immune complexes (IC): through the BCR recognizing the anti-Hsp70 IgG, and through CD91 binding Hsp70. These details were delineated in mouse experiments^48^, but their reproducibility in RA patients is unclear. A second hypothesis proposes that smoking leads to increased lung production of IgG, which would be recognized by RF in its native form and by ACPA and ACarPA as the citrullinated and carbamylated modified IgGH fragment, respectively^59^. This hypothesis has the appeal of the simplicity of considering a single protein as the link between the different RA antibodies. Accordingly, it has been characterized as the common antigen model^54^. However, only RF is known to recognize the IgGH fragment, in the form of IgGH/HLA class II complexes^60^, whereas the binding of the modified IgGH fragment by ACPA or ACarPA has not been reported. The two hypotheses could become the starting point for future experiments.

The association of smoking with RF positive RA was known before the association with the ACPA positive patients^6,7^, however, the latter displaced RF from the focus of attention^1,2^. Probably, the peculiar nature of RF has some role in this displacement. In effect, RF can be described as an antibody against IC with a role in the development of the early antibody repertoire that in the adult can be induced in the course of sustained immunological responses that include other diseases besides RA^61–63^. However, the nature of the RF in patients with RA and non-RA subjects have significant differences. In RA, the RF production is sustained and reach higher titers; also, the range of Ig V genes that are used is broader, and the response shows signs of maturation as isotype switch and mutations changing the sequence of the CDRs, characteristics that are absent or restricted in the RF of healthy subjects^62,63^. It is understood that ACPA and citrullinated proteins, or ACarPA and carbamylated proteins, are the IC recognized in RA, but other antibodies are possible as the anti-Hsp70 antibodies (in relation to smoking) and microbial antigens (including those from viruses and the mucosal microbiota). These diverse IC could act as disease triggers^48,61^. Once RF binds to the IC, the IC become larger and capable of more efficient immune stimulation. A model of RA in which two waves of IC formation, without RF and with RF, followed by complement activation and the production of inflammatory and chemotactic mediators has been proposed^61^. It is also possible that the RF-specific B cells have a critical role in the early phases of the autoimmune response before high titers of RF have been produced^63^. In effect, the RF specific B cells are abundant in healthy subjects and the only B cells capable of efficient presentation to T cells of the antigens trapped in IC^64^. These various roles of RF and the RF-specific B cells could contribute to epitope spreading and the concordance and correlation between the RA autoantibodies.

The fact that our meta-analysis did not detect any specific association of smoking with the presence of ACPA in the patients without RF (OR = 0.95, 95% CI = 0.78–1.20) does not exclude association with a subset of the ACPA^+^ patients. Examples of such subsets could be the patients with shared epitope HLA alleles or patients with heavy and current smoking. Concerning the HLA, the presence of the shared epitope is specifically associated with the presence of ACPA and there are arguments to think it could potentiate the smoking association. The most clear argument comes from a recent study by Hedstrom *et al*. showing association with ACPA^+^/RF^−^^35^, whereas previous studies failed to stratify by the two antibodies or did not find a significant association^10,11,33,34,36–39^. The other example is supported by the same recent study, which showed differential association of heavy smokers and light smokers with RA. A difference that was more marked in the ACPA^+^/RF^+^ patients, followed by the ACPA^−^/RF^+^ patients and finally the ACPA^+^/RF^−^ patients^35^. This differential association is in agreement with our results placing the concordant seropositivity at the top and RF afterwards. However, the ACPA^+^/RF^−^ patients in Hedstrom *et al*. were significantly associated with smoking and they were not in our meta-analysis.

Our study lacks healthy controls and therefore, we were restricted to comparisons between patient subsets. However, this is a minor limitation because the association of smoking with seropositive RA patients is well-established^6,7^. In addition, the study lacks information on smoking intensity and HLA alleles. These two types of information could have provided additional insight into the relationship between smoking and the different autoantibodies. In any case, a whole analysis of the potential interactions between smoking and the HLA in the three autoantibodies is not yet possible because only the HLA alleles associated with ACPA are well-defined^65^. The other antibodies, ACarPA and RF, could be specifically associated with HLA-DRB1 alleles that are not included in the shared epitope, but that have not been yet completely defined^66–68^. Finally, we should signal that the classification tree in Fig. 4 is not appropriate to separate smokers and non-smokers. This algorithm was only intended as a tool to explore the relationships between smoking and the antibodies present in the data.

In summary, we can conclude that smoking is predominantly and reproducibly associated with the triple, RF, ACPA, and ACarPA, concordant seropositive RA. A result that highlights the need to consider the mechanisms leading to concurrent seropositivity. In the patients that are not concordant for the three antibodies, smoking was exclusively associated with RF positivity in our meta-analysis. This latter association is weaker than the association with the triple concordant patients and its exclusivity needs to be replicated, ideally in studies counting with detailed information on smoking intensity. These results call for a pathogenic model that incorporates the predominant association with multiple antibodies, which could be explained by accelerated and broadened epitope spreading reflecting some of the actions of smoking on the immune system. In addition, an explanation for the particular association of smoking with the production of RF should be sought.

## Supplementary information

Supplementary Tables.
