# Publisher Correction: A predominant involvement of the triple seropositive patients and others with rheumatoid factor in the association of smoking with rheumatoid arthritis

**DOI:** 10.1038/s41598-020-75520-9

**Published:** 2020-10-22

**Authors:** Cristina Regueiro, Luis Rodriguez-Rodriguez, Raquel Lopez-Mejias, Laura Nuño, Ana Triguero-Martinez, Eva Perez-Pampin, Alfonso Corrales, Alejandro Villalba, Yolanda Lopez-Golan, Lydia Abasolo, Sara Remuzgo-Martínez, Ana M. Ortiz, Eva Herranz, Ana Martínez-Feito, Carmen Conde, Antonio Mera-Varela, Alejandro Balsa, Isidoro Gonzalez-Alvaro, Miguel Ángel González-Gay, Benjamín Fernandez-Gutierrez, Antonio Gonzalez

**Affiliations:** 1 Experimental and Observational Rheumatology and Rheumatology Unit, Instituto de Investigacion Sanitaria, Hospital Clínico Universitario de Santiago (IDIS), Santiago de Compostela, Spain; 2 Rheumatology Department, Hospital Clínico San Carlos, Instituto Investigación Sanitaria San Carlos (IdISSC), Madrid, Spain; 3 Valdecilla Biomedical Research Institute, Hospital Universitario Marqués de Valdecilla (IDIVAL), Santander, Spain; 4 Rheumatology Department, Instituto de Investigación Hospital Universitario La Paz (IDIPAZ), Madrid, Spain; 5 Rheumatology Department, Instituto de Investigación Sanitaria La Princesa, Hospital Universitario de La Princesa (IIS-lP), Madrid, Spain; 6 Immuno-Rheumatology Department, Instituto de Investigación Hospital Universitario La Paz (IDIPAZ), Madrid, Spain; 7 Faculty of Medicine and Dentistry, University of Santiago de Compostela, Santiago de Compostela, Spain

Correction to: *Scientific Reports* https://doi.org/10.1038/s41598-020-60305-x, published online 25 February 2020

In the original version of this Article, the author Miguel Ángel González-Gay was incorrectly indexed. This error has now been corrected in the HTML and PDF versions of this Article.

