# Supplementary material for: A predominant involvement of the triple seropositive patients and others with rheumatoid factor in the association of smoking with rheumatoid arthritis
Source: Sci Rep. 2020 Feb 25;10:3355. doi: 10.1038/s41598-020-60305-x (PMC7042270; doi:10.1038/s41598-020-60305-x)
Supplement: Supplementary file 1 — Supplementary Tables. [file 41598_2020_60305_MOESM1_ESM.docx]

**Supplementary material**

**A predominant involvement of the triple seropositive patients and others with rheumatoid factor in the association of smoking with rheumatoid arthritis**

**Cristina Regueiro^1^, Luis Rodriguez-Rodriguez^2^, Raquel Lopez-Mejias^3^, Laura Nuño^4^, Ana Triguero-Martinez^5^, Eva Perez-Pampin^1^, Alfonso Corrales^3^, Alejandro Villalba^4^, Yolanda Lopez-Golan^1^, Lydia Abasolo^2^, Sara Remuzgo-Martínez^3^, Ana M. Ortiz^5^**, **Eva Herranz^2^, Ana Martínez-Feito^6^, Carmen Conde^1^, Antonio Mera-Varela^1,7^, Alejandro Balsa^4^, Isidoro Gonzalez-Alvaro^5^, Miguel Ángel González-Gay^3^, Benjamín Fernandez-Gutierrez^2^, Antonio Gonzalez^1*^**

^1^Experimental and Observational Rheumatology and Rheumatology Unit, Instituto de Investigacion Sanitaria, Hospital Clínico Universitario de Santiago (IDIS), Santiago de Compostela, Spain.

^2^Rheumatology Department, Hospital Clínico San Carlos, Instituto Investigación Sanitaria San Carlos (IdISSC), Madrid, Spain.

^3^Valdecilla Biomedical Research Institute, Hospital Universitario Marqués de Valdecilla (IDIVAL), Santander, Spain.

^4^Rheumatology Department, Instituto de Investigación Hospital Universitario La Paz (IDIPAZ), Madrid, Spain.

^5^Rheumatology Department, Instituto de Investigación Sanitaria la Princesa, Hospital Universitario de la Princesa (IIS-lP), Madrid, Spain.

^6^Immuno-Rheumatology Department, Instituto de Investigación Hospital Universitario La Paz (IDIPAZ), Madrid, Spain.

^7^Faculty of Medicine and Dentistry, University of Santiago de Compostela, Santiago de Compostela, Spain.

*agmartinezp@ser.es

**Supplementary Table 1: Patient strata according to the presence of the three RA autoantibodies and the codes used for identifying them.**

**Supplementary Table S2: Lack of difference between EAC and prevalent RA cohorts in the associations of smoking with the number of autoantibodies.** Subgroup meta-analysis of the 9 available cohorts stratified in cohorts with EA patients and cohorts including patients with prevalent RA was done with Review Manager (RevMan) [Computer program]. Version 5.3. Copenhagen: The Nordic Cochrane Centre, The Cochrane Collaboration, 2014.

| Comparison | EAC | Prevalent RA | All cohorts | p for subgroup difference |
| --- | --- | --- | --- | --- |
| 1 Ab *vs.* 0 Ab | 1.10 (0.88-1.38) | 1.07 (0.80-1.43) | 1.09 (0.91-1.30) | 0.88 |
| 2 Ab *vs.* 0 Ab | 1.41 (1.13-1.74) | 1.06 (0.81-1.39) | 1.26 (1.06-1.49) | 0.11 |
| 3 Ab *vs.* 0 Ab | 2.12 (1.72-2.61) | 1.84 (1.40-2.42) | 2.01 (1.70-2.38) | 0.43 |
| 2 Ab *vs.* 1 Ab | 1.18 (0.92-1.52) | 1.10 (0.91-1.34) | 1.10 (0.91-1.34) | 0.43 |
| 3 Ab *vs.* 1 Ab | 1.81 (1.41-2.32) | 1.73 (1.28-2.34) | 1.78 (1.47-2.15) | 0.83 |
| 3 Ab *vs.* 2 Ab | 1.45 (1.16-1.82) | 1.71 (1.29-2.27) | 1.55 (1.30-1.84) | 0.37 |

**Supplementary Table S3. Association of smoking with RF-stratified patients that were positive only for one antibody ^a^.**

|  | 0 Ab | RF^+^_1_^b^ | RF^-^_1_ |
| --- | --- | --- | --- |
| IDIPAZ |  |  |  |
| Non-smoker, n | 40 | 2 | 5 |
| Smoker, n | 14 | 6 | 8 |
| PEARL |  |  |  |
| Non-smoker, n | 37 | 13 | 15 |
| Smoker, n | 22 | 10 | 7 |
| IdISSC |  |  |  |
| Non-smoker, n | 90 | 45 | 15 |
| Smoker, n | 51 | 42 | 16 |
| IDIS |  |  |  |
| Non-smoker, n | 83 | 28 | 53 |
| Smoker, n | 28 | 8 | 6 |
| IDIVAL |  |  |  |
| Non-smoker, n | 88 | 18 | 21 |
| Smoker, n | 85 | 21 | 18 |
| van Wesemael ^c^ |  |  |  |
| Non-smoker, n | 367 | 84 | 100 |
| Smoker, n | 430 | 122 | 100 |
| Rome |  |  |  |
| Non-smoker, n | 31 | 14 | 20 |
| Smoker, n | 31 | 12 | 21 |
| Summary |  |  |  |
| OR | 1 (ref.) | 1.28 | 0.93 |
| 95 % CI | - | 1.03-1.61 | 0.74-1.18 |
| p | - | 0.03 | 0.56 |
| I^2^, % | - | 16.5 | 57.4 |
| OR_re_ | 1 (ref.) | 1.29 | 1.01 |
| 95 % CI_re_ | - | 0.98-1.70 | 0.65-1.56 |
| p_re_ | - | 0.07 | 0.97 |

^a^ The table presents the number of patients in each category in the upper part and the summary statistics obtained with meta-analysis in the lower part. The triple negative patients were used as reference (ref.).

^b^ RF^+^_1_ = RF^+^ACPA^-^ACarPA^-^ patients; RF^-^_1_ = RF^-^ACPA^+^ACarPA^-^ and RF^-^ACPA^-^ACarPA^+^ patients; n = number of subjects; OR = odds ratio; CI = confidence interval; I^2^ = inconsistence; the re subscript indicates the random effects model.

^c^ No cohort-specific information was available for the three cohorts in van Wesemael *et al.*

**Supplementary Table S4. Association of smoking with RF-stratified patients that were positive for two antibodies ^a^.**

|  | 0 Ab | RF^+^_2_ | RF^-^_2_ |
| --- | --- | --- | --- |
| IDIPAZ |  |  |  |
| Non-smoker, n | 40 | 35 | 3 |
| Smoker, n | 14 | 35 | 5 |
| PEARL |  |  |  |
| Non-smoker, n | 37 | 47 | 4 |
| Smoker, n | 22 | 32 | 4 |
| IdISSC |  |  |  |
| Non-smoker, n | 90 | 69 | 6 |
| Smoker, n | 51 | 47 | 1 |
| IDIS |  |  |  |
| Non-smoker, n | 83 | 120 | 18 |
| Smoker, n | 28 | 26 | 1 |
| IDIVAL |  |  |  |
| Non-smoker, n | 88 | 35 | 6 |
| Smoker, n | 85 | 46 | 6 |
| van Wesemael ^c^ |  |  |  |
| Non-smoker, n | 367 | 134 | 37 |
| Smoker, n | 430 | 220 | 38 |
| Rome |  |  |  |
| Non-smoker, n | 31 | 43 | 2 |
| Smoker, n | 31 | 51 | 2 |
| Summary |  |  |  |
| OR | 1 (ref.) | 1.30 | 0.95 |
| 95 % CI | - | 1.09-1.55 | 0.64-1.39 |
| p | - | 0.004 | 0.78 |
| I^2^, % | - | 39.7 | 31.5 |
| OR_re_ | 1 (ref.) | 1.27 | 0.99 |
| 95 % CI_re_ | - | 0.98-1.64 | 0.54-1.80 |
| p_re_ | - | 0.07 | 0.96 |

^a^ This table follows the schema described for Supplementary Table 3.

^b^ RF^+^_2_ = RF^+^ACPA^+^ACarPA^-^ and RF^+^ACPA^-^ACarPA^+^ patients; RF^-^_2_ = RF^-^ACPA^+^ACarPA^+^ patients;; n = number of subjects; OR = odds ratio; CI = confidence interval; I^2^ = inconsistence; the re subscript indicates the random effects model.

^c^ No cohort-specific information was available for the three cohorts in van Wesemael *et al.*

**Supplementary Table S5: Lack of difference between EAC and prevalent RA cohorts in the associations of smoking with the patients bearing RF.** Analyses were done as in Table S2.

| Comparison | EAC | Prevalent RA | All cohorts | p for subgroup difference |
| --- | --- | --- | --- | --- |
| RF^+^_1_ *vs*. 0 Ab | 1.33 (0.99-1.77) | 1.24 (0.87-1.76) | 1.29 (1.03-1.61) | 0.76 |
| RF^+^_2_ *vs.* 0 Ab | 1.46 (1.16-1.84) | 1.08 (0.82-1.43) | 1.30 (1.09-1.55) | 0.10 |
| RF^+^_1+2_ *vs.* 0 Ab | 1.41 (1.16-1.72) | 1.14 (0.88-1.46) | 1.30 (1.11-1.52) | 0.19 |
| RF^+^_1+2_ *vs.* RF^+^_0+1+2_ | 1.41 (1.17-1.70) | 1.19 (0.94-1.51) | 1.32 (1.14-1.53) | 0.27 |

**Supplementary Table S6: Correlation between the titers of one antibody and the status of another**. The patients with known antibody titers for each antibody were stratified in negative , low and high and further subdivided in function of the status of a second antibody. The threshold between low and high were defined according to the ACR/EULAR 2010 classification criteria at 3 x the upper level of normal.

| Ab titer | ACPA  status | n RF | n ACarPA | RF  status | n ACPA | n ACarPA | ACarPA  status | n RF | n ACPA |
| --- | --- | --- | --- | --- | --- | --- | --- | --- | --- |
| neg | neg | 590 | 732 | neg | 590 | 649 | neg | 649 | 732 |
| low | neg | 73 | 86 | neg | 25 | 87 | neg | 130 | 81 |
| high | neg | 79 | 20 | neg | 101 | 19 | neg | 224 | 382 |
| neg | pos | 165 | 561 | pos | 244 | 641 | pos | 109 | 106 |
| low | pos | 129 | 350 | pos | 92 | 352 | pos | 72 | 38 |
| high | pos | 391 | 202 | pos | 663 | 202 | pos | 247 | 386 |
| OR |  | 4.3 | 4.4 |  | 4.1 | 3.7 |  | 2.6 | 2.6 |
| p |  | <10^-16^ | <10^-16^ |  | <10^-16^ | <10^-16^ |  | <10^-16^ | <10^-16^ |
